# Downsloping High-Frequency Hearing Loss Due to Inner Ear Tricellular Tight Junction Disruption by a Novel *ILDR1* Mutation in the Ig-Like Domain

**DOI:** 10.1371/journal.pone.0116931

**Published:** 2015-02-10

**Authors:** Nayoung K. D. Kim, Tomohito Higashi, Kyoung Yeul Lee, Ah Reum Kim, Shin-ichiro Kitajiri, Min Young Kim, Mun Young Chang, Veronica Kim, Seung-Ha Oh, Dongsup Kim, Mikio Furuse, Woong-Yang Park, Byung Yoon Choi

**Affiliations:** 1 Samsung Genome Institute, Samsung Medical Center, Seoul, Korea; 2 Department of Physiology and Cell Biology, Kobe University Graduate School of Medicine, Kobe, Japan; 3 Department of Bio and Brain Engineering, Korea Advanced Institute of Science and Technology, Daejeon, Korea; 4 Otorhinolaryngology, Seoul National University Hospital, College of Medicine, Seoul National University, Seoul, Korea; 5 Department of Otolaryngology- Head and Neck Surgery, Kyoto University Graduate School of Medicine, Kyoto, Japan; 6 Department of Otorhinolaryngology, Seoul National University Bundang Hospital, Seongnam, Korea; 7 Department of Cell and Systems Biology, University of Toronto St. George, Toronto, Canada; 8 Department of Molecular Cell Biology, Sungkyunkwan University School of Medicine, Suwon, Korea; University of South Florida, UNITED STATES

## Abstract

The immunoglobulin (Ig)-like domain containing receptor 1 (*ILDR1*) gene encodes angulin-2/ILDR1, a recently discovered tight junction protein, which forms tricellular tight junction (tTJ) structures with tricellulin and lipolysis-stimulated lipoprotein receptor (LSR) at tricellular contacts (TCs) in the inner ear. Previously reported recessive mutations within *ILDR1* have been shown to cause severe to profound nonsyndromic sensorineural hearing loss (SNHL), DFNB42. Whole-exome sequencing of a Korean multiplex family segregating partial deafness identified a novel homozygous *ILDR1* variant (p.P69H) within the Ig-like domain. To address the pathogenicity of p.P69H, the angulin-2/ILDR1 p.P69H variant protein, along with the previously reported pathogenic *ILDR1* mutations, was expressed in angulin-1/LSR knockdown epithelial cells. Interestingly, partial mislocalization of the p.P69H variant protein and tricellulin at TCs was observed, in contrast to a severe mislocalization and complete failure of tricellulin recruitment of the other reported *ILDR1* mutations. Additionally, three-dimensional protein modeling revealed that angulin-2/ILDR1 contributed to tTJ by forming a homo-trimer structure through its Ig-like domain, and the p.P69H variant was predicted to disturb homo-trimer formation. In this study, we propose a possible role of angulin-2/ILDR1 in tTJ formation in the inner ear and a wider audiologic phenotypic spectrum of DFNB42 caused by mutations within *ILDR1.*

## Introduction

Tight junctions (TJs) are specialized structures within the barrier of epithelial cells. TJs prevent the passage of some solutes through the paracellular pathway of adjacent epithelial cells [1,2]. TJs can be divided into bicellular TJs (bTJs) and tricellular tight junctions (tTJs), depending upon the location of the paracellular pathway. Freeze-fracture electron microscopy has shown that tTJs exist at tricellular contacts (TCs), where three epithelial cells meet [3,4]. At the TCs, the topmost apical bicellular TJ strands join and turn vertically in the basal direction. In turn, these strands recruit additional vertically oriented TJ strands to form a central tube structure surrounded by central sealing elements in the extracellular space at TC locations [4–7].

To date, tricellulin, angulin-1/lipolysis-stimulated lipoprotein receptor (LSR), angulin-2/immunoglobulin-like domain containing receptor 1 (ILDR1) and angulin-3/immunoglobulin-like domain containing receptor 2 (ILDR2) have been sequentially identified as protein components of tTJs [4,8,9]. Among the genes encoding these proteins, mutated *TRIC* (tricellulin) and *ILDR1* (angulin-2/ILDR1) have been reported to cause autosomal recessive nonsyndromic deafness DFNB49 (MIM 610153) [10–12] and DFNB42 (MIM 609646) [13,14], respectively. Data from experiments using epithelial cell lines with overexpressed or knocked down tricellulin suggest that these structures are responsible for epithelial barrier function, thereby controlling the paracellular flux of macromolecules [4,5,15]. Recent reports on mutated tricellulin knock-in mice, which lack tricellulin at TJs, showed disrupted bicellular and tricellular TJ strands in the inner ear epithelia and rapid progression of hearing loss accompanied by loss of cochlear hair cells [16]. Interestingly, experiments have also demonstrated that the inhibition of endolymph production in mutated tricellulin knock-in mice can rescue cochlear hair cell loss, supporting the hypothesis that defective paracellular permeability due to a dysfunctional tTJ in the inner ear could result in a toxic microenvironment in the organ of Corti [16].

In the present study, we identified a novel mutant allele of *ILDR1* (p.P69H) located in the immunoglobulin-like (Ig-like) domain through whole-exome sequencing (WES) that segregates as an autosomal recessive nonsyndromic sensorineural hearing loss (SNHL) allele in a Korean family. This novel *ILDR1* mutant allele is associated with weakened localization of angulin-2/ILDR1 at TCs, as well as a milder auditory phenotype compared with the originally reported DFNB42 profound deafness phenotype. Using protein modeling, we predict that angulin-2/ILDR1 contributes to tTJs by forming a trimer structure through its Ig-like domain, and its variant p.P69H localizes in the interface of the trimer complex to disrupt the stability of the structure.

## Materials and Methods

### Subjects and audiometric analysis

**Figure 1:**
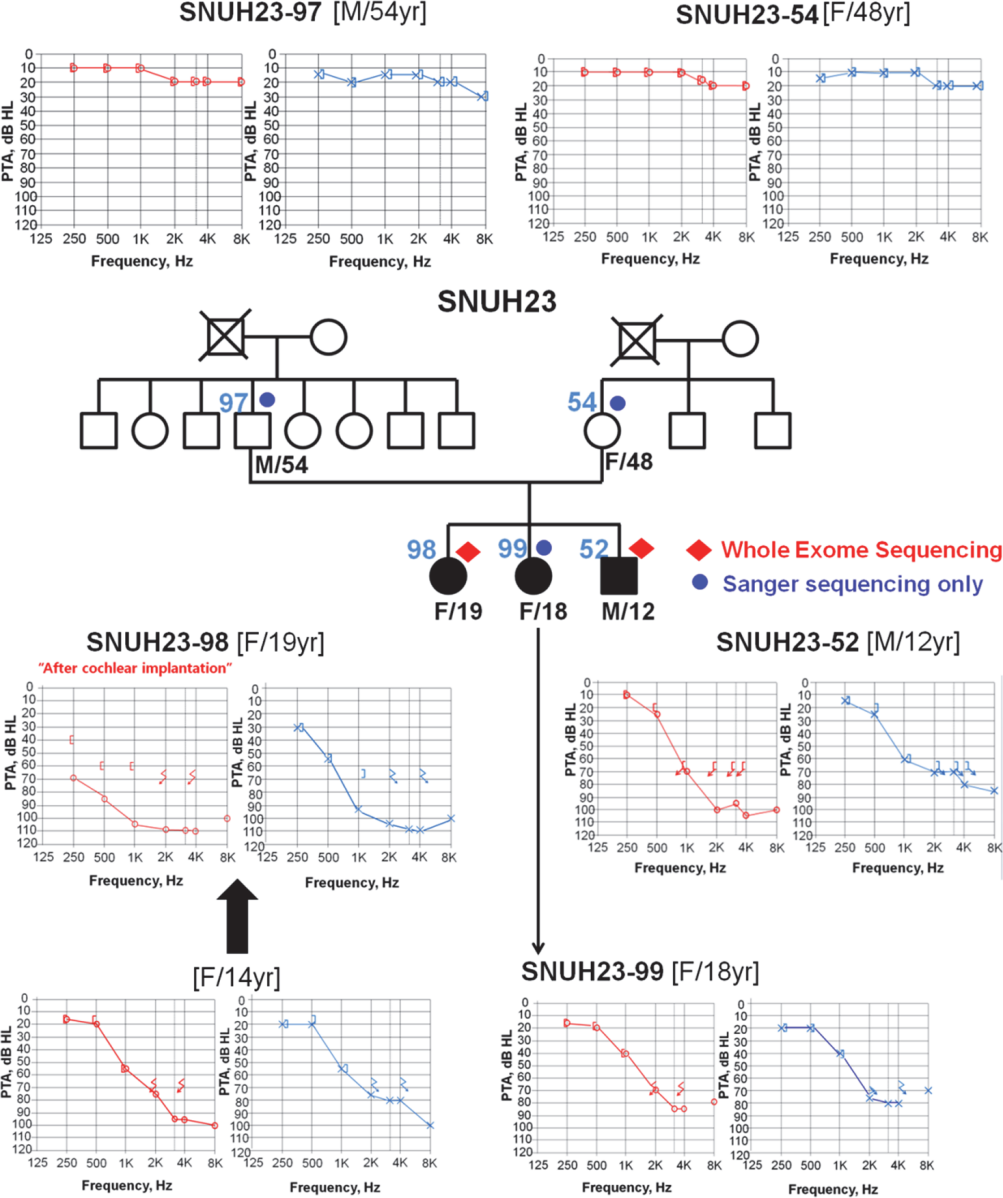
Pedigree and audiograms of SNUH23-segregating partial deafness. All three siblings (SNUH23–98, -99 and-52) exhibited partial deafness with preservation of significant low-frequency hearing thresholds. A meaningful progression of hearing loss over 5 years was observed in SNUH23–98 in the left ear (blue), whereas the right ear experienced hearing loss directly after cochlear implantation (red).

This study was approved by the Institutional Review Boards (IRBs) of Seoul National University Hospital (IRBYH-0905–041–281) and Seoul National University Bundang Hospital (IRB-B-1007–105–402). Written informed consent for participation in the study was obtained from participants or from a parent/guardian in the case of child participants (SH23–52). A three-generation pedigree was established for the family (SNUH23) (Fig. 1). Among the 16 subjects, five family members were willing to participate. DNA from blood lymphocytes was isolated from all five subjects.

Both pure tone audiometry (PTA) and physical examinations were performed on four members (Fig. 1). PTA, with air and bone conduction at frequencies ranging from 250 to 8,000 Hz, was performed on the four recruited subjects according to standard protocols. The auditory phenotype was inferred from medical and developmental history interviews with subject SH23–99.

### WES, alignment, coverage, calculation, and variant detection

WES was performed on two siblings, SH23–98 and SH23–52, at the Samsung Genome Institute. Genomic DNA was captured using the SureSelect Human All Exon Kit V3 (Agilent, Santa Clara, CA, USA) and sequenced using a HiSeq2000 system (Illumina, San Diego, CA, USA) with 101 base-paired end reads. Sequence reads were aligned with the human genome reference sequence (hg19) using BWA-v0.6.2 with the default settings. Sam tools v0.1.18, GATK v2.4–9 and Picard v1.88 were used to process SAM/BAM files, perform local realignment, and mark duplicates. Base recalibration and variant calling were performed using the GATK unified genotyper (known single nucleotide polymorphisms [SNPs] and insertions/deletions [indels] from dbSNP137, Mills and 1000G gold standard indels b37 sites, and 1000G phase1 indels b37 sites). Variants were also recalibrated using GATK based on dbSNP137, Mills indels, Hap Map, and Omni. Perl script, offered by ANNOVAR, was used to annotate the variants. Only exonic and splicing variants including non-synonymous variants and small indels were filtered. Variants with an allele frequency greater than 1% were discarded based on NHLBI-ESP 6500, 1000 Genomes Project, and an in-house database consisting of 44 Korean individuals. Of the variants identified in both siblings, only the variants that have not been reported in dbSNP137 were included. Low quality reads (< 20) and genotyping (<30) or variant/total depth < 0.2 were ruled out subsequently.

### Localization of ILDR1 at TCs

The rat anti-tricellulin antibody (Ab) used in this study has been described previously [4]. Mouse anti-FLAG monoclonal antibody (mAb), rabbit anti-claudin-3 polyclonal antibody (pAb) and Cy3-conjugated donkey anti-mouse IgG were purchased from WAKO Chemicals (Osaka, Japan), Zymed (San Francisco, CA, USA) and Jackson ImmunoResearch Laboratories (Bar Harbor, ME, USA), respectively. Alexa 488-conjugated donkey anti-rat IgG and Alexa 647-conjugated donkey anti-rabbit IgG Molecular Probes were obtained from Life Technologies (Eugene, OR, USA).

Mouse mammary epithelial EpH4 cells were cultured in Dulbecco’s Modified Eagle Medium (DMEM) supplemented with 10% fetal calf serum (FCS). The knockdown of angulin-1/LSR in EpH4 cells and the cDNA sequence encoding human angulin-2/ILDR1 have been described previously [8]. The p.P69H mutation was introduced by site-directed mutagenesis. DNA fragments encoding angulin-2/ILDR1 were subcloned into the pCAG-cFLAG2 vector. To evaluate the tricellulin-recruiting property of human angulin-2/ILDR1, angulin-1/LSR knockdown EpH4 cells were transfected with plasmids encoding either wild-type human angulin-2/ILDR1, p.P69H, p.R97Q, p.Q195X, or a mock plasmid, and stable clones were selected using G418 added to the medium.

For immunofluorescent staining, each clone was cultured on coverslips, fixed with 1% formaldehyde in phosphate-buffered saline (PBS), blocked with 2% bovine serum albumin (BSA) in PBS, and incubated with the above-described primary and secondary antibodies. After washing with PBS, samples were embedded in FluorSave reagents (Calbiochem, San Diego, CA, USA) and observed under a fluorescence microscope (IX71; Olympus, Tokyo, Japan) equipped with a cooled charge-coupled device camera (ORCA-ER; Hamamatsu Photonics K. K., Hamamatsu City, Japan) controlled by Power Macintosh G5 and IPLab V3.9.5 software (BD Biosciences, San Jose, CA, USA) at room temperature.

### Three-dimensional protein modeling of the Ig-like domain

**Figure 2:**
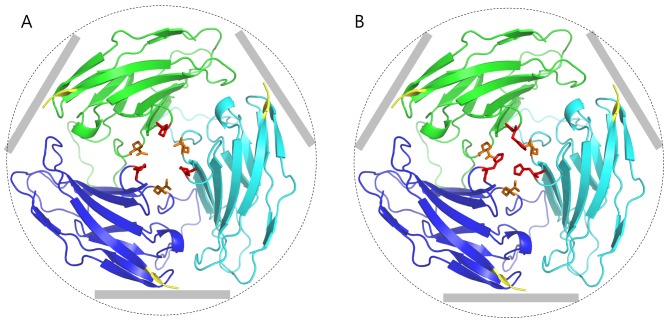
Trimer structure model of ig-like domains of ILDR1. The red and orange residues are the 69th and 97th residue, respectively. The cell membrane of each epithelial cell is located after the C-terminus (red color). The grey bars schematically represent the cell membranes surrounding the tricellular junction (dashed circle). Both 69th residues and 97th residues are gathered near interface region, which explains why p.P69H and p.R97Q mutations weakening the localization. (A) wild type trimer (B) p.P69H mutant trimer.

To obtain structural insight regarding the effects of the p.P69H mutation on tricellular localization of the angulin-2/ILDR1 protein, a structural model of the Ig-like domain of angulin-2/ILDR1 was constructed. Since the Ig-like domain is located within the extracellular domain at the site where the mutation occurs, only the Ig-like domain was modeled rather than the entire protein. First, we identified feasible template structures for the Ig-like domain of angulin-2/ILDR1 from its homologs using BLAST (http://blast.ncbi.nlm.nih.gov/Blast.cgi) and HHsearch (http://toolkit.tuebingen.mpg.de/hhpred). Among these structures, 1XT5 (crystal structure of VCBP3, domain 1, from Branchiostomafloridae), which demonstrated the highest alignment score, was chosen as the template structure. The three-dimensional (3D) structural model for a single Ig-like domain of angulin-2/ILDR1was then constructed using Modeller (http://salilab.org/modeller/) (S1 Fig.). In addition, loop structures within the model were refined using Rosetta loop modeling (https://www.rosettacommons.org/). To assess the physiochemical impact of the p.P69H mutation, we built a complex structural model of the Ig-like domains of angulin-2/ILDR1, assuming that the complex of Ig-like domains of angulin-2/ILDR1 formed a homo-trimeric structure based on their colocalization at the extracellular region of three neighboring epithelial cells [8]. First, 50,000 symmetric trimer models of angulin-2/ILDR1 Ig-like domains were built using Rosetta Dock (https://www.rosettacommons.org/). These structures were then ranked by their interface energy score. After choosing the upper 500 (1%) structures, the most plausible trimeric structure was then selected as the final trimer model. The criteria used were as follows: (i) the putative membrane location for each chain was plausible (i.e., the C-terminus of each domain was not buried inside the complex), (ii) the interface energy score, and (iii) the number of polar interactions between interface residues capable of forming hydrogen bonds. The mutant (p.P69H) trimer model was then constructed from the wild-type trimer model using Fold X (http://foldx.crg.es/) (Fig. 2). A molecular dynamics (MD) simulation was also performed using Gromacs (http://www.gromacs.org/) to compare the dynamic stability of wild-type and mutant trimer models to ultimately assess the impact of the p.P69H mutation.

## Results

### Clinical features of the NSHL family

PTA was performed on five family members, three of whom (SH23–98, SH23–99 and SH23–52) exhibited a post-lingual onset and partial deafness (Fig. 1). SH23–98 manifested significant progression of hearing loss over 5 years, and parents recalled that SH23–99 and SH23–54 had near-normal hearing and speech development until 10 years of age. Three members (SH23–98, SH23–99 and SH23–52) demonstrated normal cognitive function in the absence of any anomalous-looking features indicative of syndromic hearing loss. Detailed imaging studies, including brain magnetic resonance imaging and temporal bone computed tomography scans, did not reveal any inner ear abnormality. Both parents (SH23–97 and SH23–54) showed normal hearing, indicating autosomal recessive hearing loss in this family (Fig. 1). Since *TMPRSS3*, the gene encoding transmembrane protease serine 3, is the most frequently detected gene in subjects with ski-slope type hearing loss in the Korean population [17], we examined the *TMPRSS3* sequence present in SH23–98 and SH23–52 to identify any mutation(s) located within the exons and/or splice junctions; however, we failed to identify any mutations within this gene.

We have also performed a rigorous systemic physical examination to detect, if any, subtle abnormalities relevant to the recently proposed role of angulin-2/ILDR1 [18], however, we failed to identify any other abnormality than partial deafness.

### WES and data analysis

**Figure 3:**
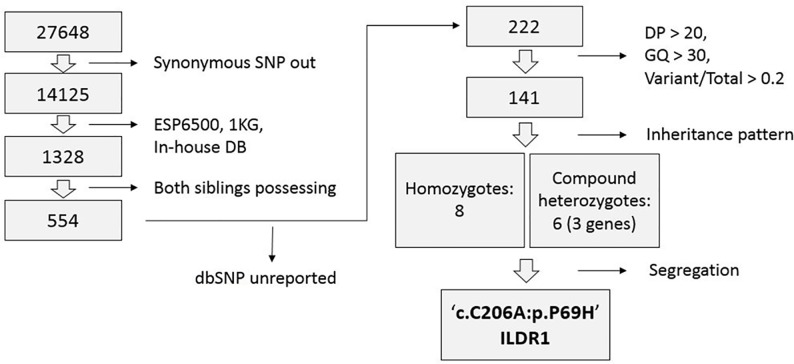
Filtering and prioritization steps for novel variant identification. Of the 27,648 variants identified in two siblings, all synonymous SNPs were excluded, and only rare variants (< 1%) based on NIHBL-ESP6500, the 1000 Genomes Project and our in-house database (44 Koreans) were included for subsequent analysis. Only variants that have not been reported in dbSNP137 were selected. Low-quality read depth (DP) and genotype (GP) under 20 and 30, respectively, or variant/total depth less than 0.2 were also discarded. Under the recessive model, eight variants of homozygotes and six variants of compound heterozygotes (11 genes in total) were identified. Further filtering of these variants was performed by Sanger sequencing and by confirming co-segregation with the phenotype. The remaining variants were also cross-checked against dbSNP138 to further exclude the irrelevant variants to the phenotype.

**Figure 4:**
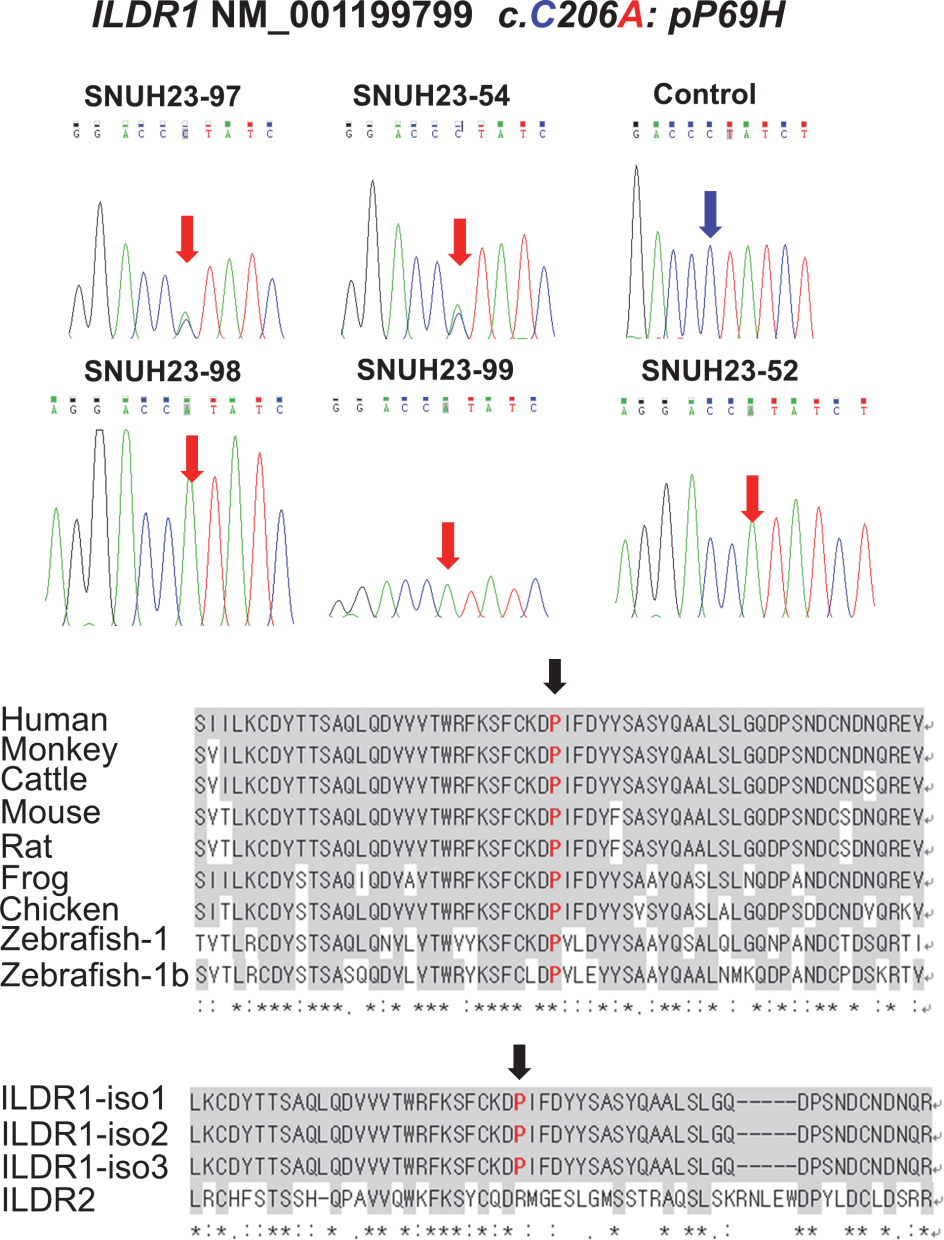
Validation of variants within *ILDR1* using Sanger sequencing. (A) Sanger sequencing trace of p.P69H within *ILDR1* from SNUH23. (B) Conservation of the p.P69 residue among orthologs and paralogs of ILDR1.

The whole exomes of two siblings (SH23–98 and SH23–52) with hearing loss were sequenced; the mean depth of the target regions was greater than 80× coverage, with nearly 90% with at least 10× coverage (S1 Table). To identify causal variants, 27,648 common single nucleotide variants and indels were filtered and prioritized in two family members (Fig. 3). Of the 141 variants, eight homozygous and six compound heterozygous variants located in 11 genes were selected under the recessive model (Table 1). The 14 variants were filtered further by Sanger sequencing and segregation analysis within the family members. Three variants from two genes (*MUC12* and *CHD3*) were excluded by Sanger sequencing. An additional four variants from two genes (*BRCA1* and *ABCA10*) did not co-segregate with the auditory phenotype among the remaining family members. Four homozygous variants from *ZSCAN26*, *NR1H2*, *VSIG10L*, and *PLK1S1* were also excluded, since they were recently reported as polymorphisms (rs61622742, rs34296657, rs11402251, and rs11087346, respectively) in dbSNP138. Finally, *MICA* and *ATXN3* variants were detected in three and four unrelated Korean normal hearing controls, respectively, of 276 chromosomes, suggesting that these variants are Korean-specific polymorphisms. Finally, only one novel homozygous missense variant, c.C206A in *ILDR1*, resulting in the substitution of p.P69H, remained and was validated by Sanger sequencing. This variant was found in a partially deafened SH23–99 (Fig. 4). The novel variant p.P69H is described as ‘damaging’ by SIFT and PolyPhen2 (0.03 and 1, respectively) and is well-conserved in various species based on the GERP++ score (5.64) (Table 2). This variant was not detected among 476 chromosomes from unrelated Korean normal hearing controls.

**Table 1:** List of final candidate SNVs in two siblings with hearing loss.

Inheritance	Gene	Func	GenbankID	Exon	Nucleotide	AA	Chr	Start	End	Ref	Alt	SH23_52		SH23_98	
												DP	GQ	DP	GQ
Homo	ILDR1	NS	NM_001199799	Exon2	c.C206A	p.P69H	chr3	121725861	121725861	G	T	25	75	40	99
Homo	ZSCAN26	-	-	-	-	-	chr6	28239932	28239932	-	G	28	84	40	78
Homo	MICA	FI	NM_000247	exon6	c.949–1->CTGCTGCTGCT	NA	chr6	31380161	31380161	-	CTGCTGCTGCT	23	66	24	72
Homo	MUC12	NS	NM_001164462	exon2	c.C7682T	p.T2561M	chr7	100641526	100641526	C	T	203	99	238	99
Homo	ATXN3	NI	NM_001164782	exon2	c.68_69insAGCAGCAGCAGC	p.G23delinsGAAAA	chr14	92537353	92537353	-	GCTGCTGCTGCT	50	99	66	99
Homo	NR1H2	NI	NM_001256647	exon5	c.225_226insCAG	p.K75delinsKQ	chr19	50881822	50881822	-	CAG	64	99	73	99
Homo	VSIG10L	FI	NM_001163922	exon10	c.2577dupC	p.A859fs	chr19	51835892	51835892	-	G	47	99	72	99
Homo	PLK1S1	-	-	-	-	-	chr20	21186161	21186161	-	G	67	99	96	99
Com het	CHD3	NS	NM_001005271	exon5	c.G898C	p.A300P	chr17	7796815	7796815	G	C	60	99	84	99
		NS	NM_001005271	exon5	c.T902C	p.L301P	chr17	7796819	7796819	T	C	70	87	102	99
Com het	BRCA1	NS	NM_007297	exon9	c.T1789A	p.C597S	chr17	41245618	41245618	A	T	138	99	206	99
		NS	NM_007297	exon3	c.C13T	p.L5F	chr17	41258531	41258531	G	A	52	99	61	99
Com het	ABCA10	NS	NM_080282	exon32	c.C3779T	p.A1260V	chr17	67150383	67150383	G	A	107	99	158	99
		NS	NM_080282	exon32	c.G3778C	p.A1260P	chr17	67150384	67150384	C	G	109	99	162	99

Candidate variants were identified under the recessive model (either homozygotes or compound heterozygotes). AA: Amino Acid; Ref: Reference; Alt: Alternative; NS: Nonsynonymous SNV; FD: Frameshift Deletion; FI: Frameshft Insertion; NI: Nonframeshift Insertion; DP: Depth; GQ: Genotype Quality; Homo: Homozygotes; Com het: Compound heterozygotes.

**Table 2:** Causal variant *ILDR1*.

Gene	Patient	Inheritance	DP	GQ	Type	Genomic location	Genbank No.	Exon	Nucleotide	Protein	R	A	ESP6500	1000G	dbSNP137	SIFT	PolyPhen2 (HVAR)	GERP++
ILDR1	SH23_52	homo	25	75	nonsynonymous SNV	chr3:121725861	NM_001199799	exon2	c.C206A	p.P69H	G	T	-	-	-	0.03	1	5.64
	SH23_98	homo	40	99														

R: Reference; A: Alternative; DP: Depth; GQ: Genotype Quality.

### Localization of angulin-2/ILDR1 at TCs

**Figure 5:**
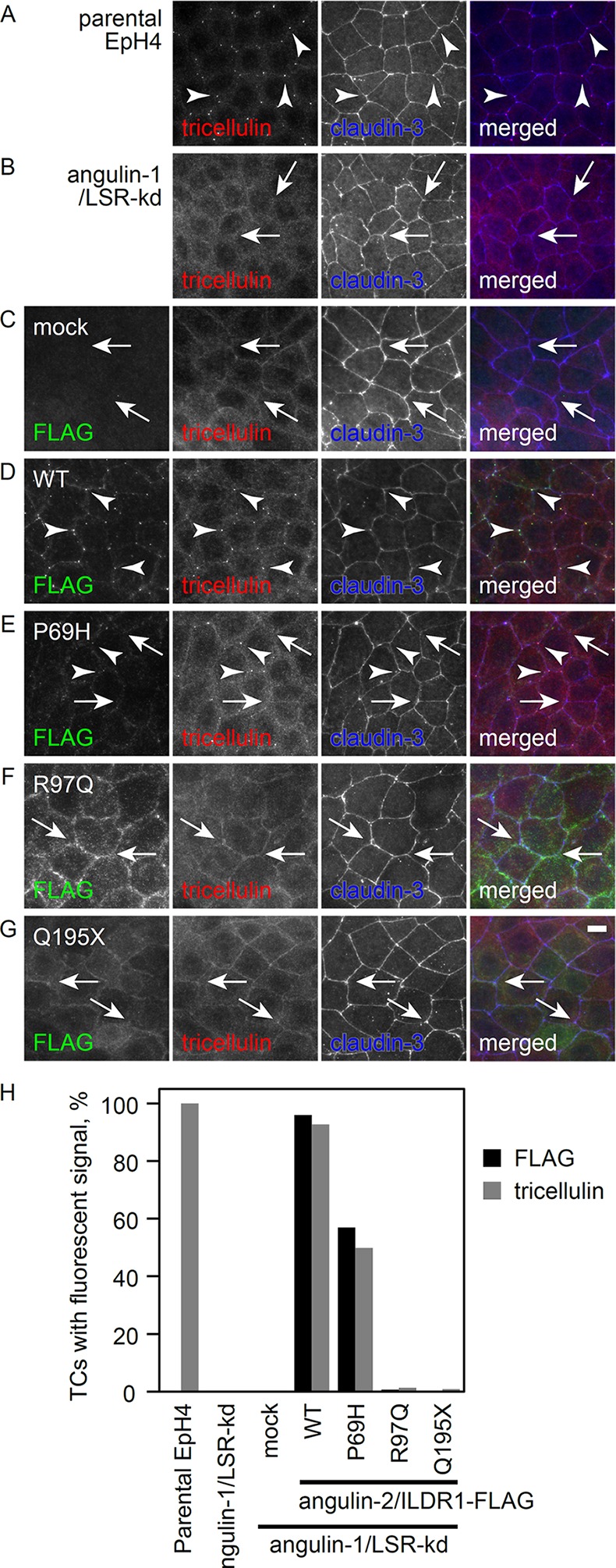
Effect of the p.P69H mutation on the localization and tricellulin recruitment function of ILDR1. (A-G) Parental EpH4 cells, (A) LSR knockdown cells. (B) LSR knockdown cells stably expressing the mock FLAG vector (C) or wild-type (WT) human ILDR1. Immunostaining of (D) p.P69H, (E) p.R97Q, (F) or p.Q195X (G) was performed using anti-FLAG mAb (green), anti-tricellulin mAb (red) and anti-claudin-3 pAb (blue). Arrowheads and arrows indicate tricellular contacts with and without tricellulin localization, respectively. Scale bar: 10 μm. (H) Statistical analysis of FLAG and tricellulin localization at TCs. More than 600 TCs for each cell clone were examined for FLAG and tricellulin accumulation.

Most, if not all, angulin-2/ILDR1 protein-containing mutations reported to date in DFNB42 patients exhibit failure to localize at TCs and defectiveness in tricellulin recruitment in angulin-1/LSR knockdown EpH4 cells [8]. Therefore, we determined whether the p.P69H mutation affects the localization of angulin-2/ILDR1 or tricellulin recruitment. As reported previously, tricellulin was mislocalized to the lateral membrane in angulin-1/LSR knockdown EpH4 cells, in contrast to its normal localization at TCs in parental EpH4 cells (Fig. 5A and 5B). The exogenously expressed wild-type angulin-2/ILDR1 was nearly always localized at TCs and able to recruit tricellulin to TCs (Fig. 5D). On the other hand, the p.P69H mutant was concentrated at approximately half of the TCs of angulin-1/LSR knockdown cells. These data indicate that the p.P69H mutation affects the ability of angulin-2/ILDR1 to localize at TCs (411/722 for p.P69H vs. 593/618 for wild-type; *p* = 1.14 x 10^–60^) (Fig. 5E and 5H). Tricellulin was also concentrated at approximately half of all TCs in p.P69H-expressing angulin-1/LSR knockdown cells (360/722 for P69H vs. 573/618 for wild-type; *p* = 7.34 x 10^–65^) (Fig. 5D and 5H). p.R97Q and p.Q195X mutants, which served as negative controls, were not concentrated at TCs and showed no involvement in tricellulin recruitment (Fig. 5F, 5G, and 5H). These results indicate that the p.P69H mutation significantly affects the localization of angulin-2/ILDR1, although the extent of mislocalization was less severe than that of the p.R97Q and p.Q195X mutations. Therefore, the difference in the deafness phenotype may reflect the severity of mislocalization.

### 3D protein modeling of the Ig-like domain

The localization of angulin-2/ILDR1 at TCs was reported recently [8]. In the study described herein, the p.P69H mutation was observed in our patients with partial deafness. These data suggest that the Ig-like domain of angulin-2/ILDR1, where p.P69H resides, may be involved in the localization of angulin-2/ILDR1 at TCs. However, the mechanism of localization is not clear. It has yet to be determined whether angulin-2/ILDR1 is able to localize at TCs without assistance from other protein(s). Most Ig-like domains, such as that in angulin-2/ILDR1, are important in cell adhesion [9,19]. Therefore, we hypothesized that angulin-2/ILDR1 may function by forming direct interactions with tTJ proteins. Thus, we plan to study the interaction between angulin-2/ILDR1 and other components that drive the localization of angulin-2/ILDR1 at TCs by modeling their complex structure, although any additional components have yet to be elucidated. Correct localization of angulin-2/ILDR1 at TCs was also observed in Tric^R497X/R497X^ mice lacking tricellulin at TCs, indicating that tricellulin does not drive angulin-2/ILDR1 localization [16]. We further believe that angulin-2/ILDR1 may be the main driver of localization, and three TCs from each cell could form a trimer to initiate the tTJ. In order to test this hypothesis, we constructed a 3D model of the angulin-2/ILDR1 trimer structure (Fig. 2). In this model, the p.P69H mutation and the previously reported deleterious p.R97Q mutation [13] were both located at the interface of the complex, explaining why these mutations weaken its localization. The pathogenicity of the p.P69H mutation can be predicted by the trimer model. Since residue 69 was mutated from proline to histidine, the loop conformation may have been shifted due to differences in flexibility. Additionally, the space for placement of residue 69 is narrow. Since histidine has a larger side chain than proline, repulsive forces may be increased in the mutant. Moreover, unlike proline, histidine can exist in a positively charged form (~10% at physiological pH), which can further amplify the repulsion.

To further evaluate the stability of the model and to evaluate the mutational effect in greater detail, we performed molecular dynamics (MD) simulations. MD simulations were executed a total of eight times (four wild-type, four mutant). We found that the root-mean-square deviation (RMSD) of residue 69 converged to ~5 Å in the wild-type trimer model, but the RMSD in the p.P69H mutant trimer model did not converge, varying between 3~7 Å (S2 Fig.). This result indicates that the space around residue 69 can become unstable as a result of the p.P69H mutation.

## Discussion

Systematic exclusion of the variants either in the homozygous or compound heterozygous state from 10 autosomal genes based upon a segregation study among family members of SNUH23 indicated that the p.P69H allele of *ILDR1* most likely accounts for the partial deafness in this family. Less severe, but significant mislocalization of the p.P69H substitution of angulin-2/ILDR1 was noted in angulin-1/LSR knockdown EpH4 cells, further supporting its pathogenic potential. Recently, WES contributed significantly to the detection of a series of new deafness genes [20–27]. Although the SNUH23 family was not a consanguineous family and did not have a significant linkage interval prior to this study, unlike those in other studies, the presence of three affected siblings enabled detection of a causative gene through WES, coupled with segregation analysis.

A ski-slope type sensorineural hearing loss, now referred to as ‘partial deafness’, characterized by an abrupt downsloping from mild to severe/profound hearing loss across high frequencies, is a challenge for conventional auditory rehabilitation methods, such as hearing aids or cochlear implants. Based upon the progression of hearing loss and the degree of residual low-frequency hearing, appropriate auditory rehabilitation varies from patient to patient. However, the molecular etiology underlying this special configuration of hearing loss has not been elucidated, with the exception of the previously identified recessive mutations within the *TMPRSS3* gene [28,29]. Depending upon the proteolytic activity of the mutant TMPRSS3 protein [17,30], two discrete types of phenotypes can manifest. In this study, we showed that mutations within *ILDR1* can also manifest as discrete, audiologic phenotypes depending upon the degree of localization of angulin-2/ILDR1 at the TC.

Angulin-2/ILDR1 protein, a component of tTJs, provides a strong barrier property to the epithelium in cultured epithelial cells. The majority of DFNB42-associated mutations within the angulin-2/ILDR1 protein did not localize at TCs in the angulin-1/LSR knockdown epithelial cells [8]. Higashi et al. showed that angulin-2/ILDR1 interacts with tricellulin and recruits tricellulin at TCs in LSR knockdown cells, proposing the failure of angulin-2/ILDR1-mediated tricellulin recruitment as a possible pathogenesis of DFNB42 deafness. Consistent with our previous results [8], partial mislocalization of the p.P69H *ILDR1* mutant in this study led to the incomplete recruitment of tricellulin to TCs in LSR knockdown epithelial cells. This incomplete recruitment of tricellulin due to partial mislocalization of angulin-2/ILDR1 could be a direct cause of progressive partial deafness in the patients described in this study since mislocalization of tricellulin causes deafness in humans and mice regardless of angulin-2/ILDR1 [11,16]. The correlation between the degree of localization of angulin-2/ILDR1 and tricellulin at TCs in angulin-1/LSR knockdown EpH4 cells strongly supports the hypothesis that angulin-2/ILDR1 interacts directly with tricellulin.

However, there may be another mechanism for hearing loss due to alteration of angulin-2/ILDR1 *in vivo*. Recently, Morozko et al. proposed that angulin-2/ILDR1 was not required for initial recruitment of tricellulin to TCs *in vivo*. Their data indicated that failure in recruitment of tricellulin itself does not account for the early- onset severe hearing loss in DFNB49 and in angulin-2/ILDR1 deficient in mice. They observed gradual mislocalization of tricellulin at TCs after the first postnatal week in angulin-2/ILDR1 deficient mice [31]. These *in vivo* results contradict our *in vitro* findings showing that tricellulin localization was tightly correlated with that of angulin-2/ILDR1 in angulin-1/LSR knockdown EpH4 cells. The presence of other tight junction proteins *in vivo*, such as angulin-1/LSR and occludin could contribute to this discrepancy; LSR is co-expressed with angulin-2/ILDR1 in the inner ear [8], and occludin is involved in tricellulin localization [9,32]. Therefore, angulin-1/LSR or others that were lacked by our *in vitro* system may be able to recruit tricellulin to the TCs in the inner ear in the absence of angulin-2/ILDR1 expression. Given these *in vivo* findings, it is also possible that the mutation within *ILDR1* may lead to deafness via a different mechanism unrelated to failed tricellulin recruitment at TCs, or through a combination of these mechanisms. Rather, we proposed a direct effect of *ILDR1* alterations upon the tTJ structure irrespective of the status of tricellulin recruitment, based upon our protein modeling of the Ig-like domain. In fact, the position of the p.P69H mutation within the Ig-like domain and the instability of the predicted homo-trimer complex of angulin-2/ILDR1 caused by this mutation suggest that p.P69H may exert pathogenicity irrespective of the failure to recruit tricellulin at TCs. Specifically, the Ig-like domain of angulin-2/ILDR1 shares the same motif as other cell adhesion molecules (CAM) [9,19], indicating that the Ig-like domain of angulin-2/ILDR1 may attach to other proteins or the extracellular matrix (ECM) to localize proteins at TCs. In this study, we assumed that the Ig-like domain of angulin-2/ILDR1 was bound to the same type of molecule (homophilic CAM) for the following reasons. First, when a tTJ is formed, the tricellular corner would become highly narrow due to the tight junctions between epithelial cells (3~12 nm) [33]. It is difficult to expect any ECM binding motifs in this narrow space. Secondly, if CAM binding occurs via an alternative mechanism for angulin-2/ILDR1 localization, those CAMs must also be localized at the TCs. Such localized CAMs have not been found at the tricellular corner as of yet, and moreover such a mechanism seems inefficient considering that angulin-2/ILDR1 is the key protein that recruits tricellulin to the TC [8]. If we assume that the Ig-like domain of angulin-2/ILDR1 is a homophilic CAM, then a trimer would be the most plausible minimum-sized complex. Therefore, the homo-trimeric ILDR1 complex is the most practical model to currently explain angulin-2/ILDR1 localization. The lateral size of the trimer model (Fig. 2A) is 4.21 nm, which does not contradict the known size of tight junctions. Evaluation of *ILDR1* knockout or knock-in mice of a specific *ILDR1* mutation, as well as further biostructural studies, will be needed to address this issue. Indeed, freeze-fracture electron microscopy has revealed alteration of the ultrastructure of inner ear tTJ from angulin-2/ILDR1 deficient mice [31].

Results from our study are intriguing, in that a milder auditory phenotype characterized by a ‘partial deafness’ was observed in all three siblings from this family. A higher degree of hearing loss with a flat or slightly downsloping configuration was the main audiological feature of the DFNB42 family [13]. What accounts for this partial deafness? To address this question, it is important to note that partial mislocalization of angulin-2/ILDR1 at TCs was reported previously for the three *ILDR1* variants (p.Glu379X, p.Thr345ProfsX20 and p.Glu394SerfsX15) [8], albeit the degree of mislocalization of the three mutants appeared to be more severe than that of p.P69H. All *ILDR1* variants reported thus far, including the three variants described herein, are associated with severe or a more severe degree of flat-type hearing loss [8,13,14]. In this sense, the p.P69H mutant protein was the first and only variant that showed both weakened localization at TCs of angulin-1/LSR knockdown cells and a milder auditory phenotype ‘partial deafness’. It is possible that the degree of weakened localization of angulin-2/ILDR1 protein at TCs is correlated with the severity of the initial auditory phenotype. Examination of Tric^R497X/R497X^ mice, which lack tricellulin at TCs at P16, revealed that cochlear hair cell degeneration was more pronounced in the mid and basal turns compared with the apical turn [16], suggesting that the reticular lamina of the organ of Corti in the mid and basal turn is more sensitive to various types of continually varying mechanical stressors in the disrupted tTJ, thereby giving rise to a ski-slope type of partial deafness.

Alternatively, mutations within the Ig-like domain of *ILDR1* could result in the auditory phenotype that is more pronounced in mid and high frequencies. Disruption of the tTJ, due to abnormal angulin-2/ILDR1, may damage the organ of Corti preferentially in the mid and basal turn of the cochlea. To date, only three *ILDR1* variants, including p.P69H, have been reported to reside in this domain. The p.Asn109_Pro111dup mutation, reported in a consanguineous Saudi family, was associated with a non-progressive downsloping audiogram from severe to profound across higher frequencies. The residual low-frequency hearing in this Saudi family was not as significant as that in our study [14]. With respect to p.R97Q, another variant in this domain [13], the detailed audiogram of a homozygous carrier of p.R97Q has not yet been reported, precluding any genotype-phenotype correlation.

It should also be noted that significant progression of hearing loss was observed in homozygous carriers of p.P69H in this study. This is in contrast to a previous description in which DFNB42 deafness was characterized by bilateral non-progressive moderate to profound sensorineural hearing impairment [13]. This worsened hearing threshold in homozygous carriers of p.P69H within *ILDR1* in this study was compatible with aggravated hearing thresholds in a mouse model lacking tricellulin (Tric^p.R497X/p.R497X^ mice) [16]. It has been suggested that hearing loss due to impaired TJs is caused by defective permeability via the paracellular pathway, which would expose the sensory epithelium to a toxic environment [16]. Significant residual hearing at low frequencies and progressiveness never reported before with other *ILDR1* mutations suggest that the pathogenic mechanism of p.P69H may differ from those of other variants. It may be that the p.P69H mutation in the Ig-like domain affects TJ structure in the inner ear to the extent that the inner ear sensory epithelium develops normally and can be maintained for a while, but subsequently begins to degenerate due to a toxic microenvironment in the extracellular space of the hair cells especially from the basal turns. In addition, this progression should be considered for appropriate auditory rehabilitation of partial deafness.

In this study, we reported a novel missense mutation within the *ILDR1* gene associated with partial deafness, and identified partial mislocalization of the mutant angulin-2/ILDR1 protein at TCs. More importantly, we have proposed a new pathogenic mechanism of *ILDR1* mutations residing in the Ig-like domain, based upon the calculated effect of our novel *ILDR1* mutation on the stability of a predicted trimer model.

## Supporting Information

S1 Fig 3D structural model of the Ig-like domain of ILDR1. This model used 1XT5.pdb (Crystal Structure of VCBP3, domain 1, from Branchiostoma floridae) as the template structure. The red and orange residues are the 69th and 97th residue, respectively. Those two residues are the sites for the mutation p.P69H and p.R97Q, respectively.
(TIF)

S2 Fig The RMSD deviation of a wild type trimer model and a p.P69H mutant model obtained by molecular dynamics (MD) simulation. The MD simulations executed 8 times; 4 times for a wild type model (wt1~wt4) and 4 times for a mutant model (m1~m4). The RMSD values tend to converge on around 5 Å for the wild type model, while they tend to widely vary between 3~7 Å for the mutant model.
(TIF)

S1 Table Summary statistics for WES of two siblings with hearing loss. Statistical analysis was performed using Picard with the module ‘CalculateHsMetrics’. ‘UNIQUE READS’ represent the number of reads not marked as duplicates. ‘UQ READS ALIGNED’ represent the number of unique reads that align with a mapping score > 0 to the reference. ‘ON+NEAR BAIT’ represents the percentage of on + near bait bases against bases aligned, and ‘OFF BAIT’ represents the percentage of aligned bases that mapped neither on nor near a bait. ‘ZERO CVG TARGETS PCT’ represents the number of targets that did not reach coverage = 2 over any base. PCT: percent, UQ: unique, CVG: coverage.
(DOCX)
