# Supplementary figures and images for: Downsloping High-Frequency Hearing Loss Due to Inner Ear Tricellular Tight Junction Disruption by a Novel ILDR1 Mutation in the Ig-Like Domain
Source: PLoS One. 2015 Feb 10;10(2):e0116931. doi: 10.1371/journal.pone.0116931 (PMC4323246; doi:10.1371/journal.pone.0116931)

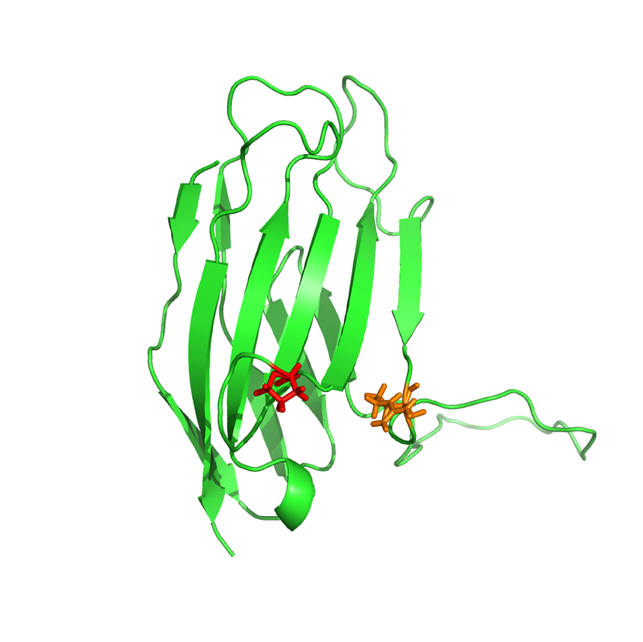

Supplement: S1 Fig — The red and orange residues are the 69th and 97th residue, respectively. Those two residues are the sites for the mutation p.P69H and p.R97Q, respectively. (TIF) [file pone.0116931.s001.tif]

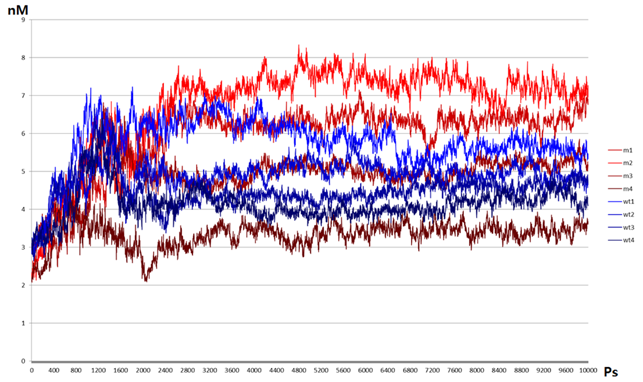

Supplement: S2 Fig — The MD simulations executed 8 times; 4 times for a wild type model (wt1~wt4) and 4 times for a mutant model (m1~m4). The RMSD values tend to converge on around 5 Å for the wild type model, while they tend to widely vary between 3~7 Å for the mutant model. (TIF) [file pone.0116931.s002.tif]
