# Supplementary material for: Downsloping High-Frequency Hearing Loss Due to Inner Ear Tricellular Tight Junction Disruption by a Novel ILDR1 Mutation in the Ig-Like Domain
Source: PLoS One. 2015 Feb 10;10(2):e0116931. doi: 10.1371/journal.pone.0116931 (PMC4323246; doi:10.1371/journal.pone.0116931)
Supplement: S1 Table — Statistical analysis was performed using Picard with the module ‘CalculateHsMetrics’. ‘UNIQUE READS’ represent the number of reads not marked as duplicates. ‘UQ READS ALIGNED’ represent the number of unique reads that align with a mapping score > 0 to the reference. ‘ON+NEAR BAIT’ represents the percentage of on + near bait bases against bases aligned, and ‘OFF BAIT’ represents the percentage of aligned bases that mapped neither on nor near a bait. ‘ZERO CVG TARGETS PCT’ represents the number of targets that did not reach coverage = 2 over any base. PCT: percent, UQ: unique, CVG: coverage. (DOCX) [file pone.0116931.s003.docx]

**Table S1.** Summary statistics for WES of two siblings with hearing loss.

| Patients | **SH23-52** | **SH23-98** |
| --- | --- | --- |
| **TOTAL READS** | **105420016** | **119480074** |
| UNIQUE READS | 72609029 | 108881707 |
| PCT UQ READS | 0.688759 | 0.911296 |
| UQ READS ALIGNED | 65514652 | 100327739 |
| PCT UQ READS ALIGNED | 0.902293 | 0.921438 |
| On + Near BAIT | 0.861511 | 0.853418 |
| OFF BAIT | 0.138489 | 0.146582 |
| **TARGET COVERAGE** | 88.497236 | 134.355788 |
| ZERO CVG TARGETS PCT | 0.032752 | 0.032156 |
| PCT TARGET BASES 10X | 0.899386 | 0.91555 |
| PCT TARGET BASES 20X | 0.836867 | 0.87379 |
| PCT TARGET BASES 30X | 0.772004 | 0.834983 |
| PCT TARGET BASES 40X | 0.702573 | 0.795265 |
| PCT TARGET BASES 50X | 0.630094 | 0.753525 |
| PCT TARGET BASES 100X | 0.316713 | 0.52826 |

Summary statistics for WES of two siblings with hearing loss. Statistical analysis was performed using Picard with the module ‘CalculateHsMetrics’. ‘UNIQUE READS’ represent the number of reads not marked as duplicates. ‘UQ READS ALIGNED’ represent the number of unique reads that align with a mapping score > 0 to the reference. ‘ON+NEAR BAIT’ represents the percentage of on + near bait bases against bases aligned, and ‘OFF BAIT’ represents the percentage of aligned bases that mapped neither on nor near a bait. ‘ZERO CVG TARGETS PCT’ represents the number of targets that did not reach coverage = 2 over any base. PCT: percent, UQ: unique, CVG: coverage.
